# Microplastic contamination of the drilling bivalve *Hiatella arctica* in Arctic rhodolith beds

**DOI:** 10.1038/s41598-021-93668-w

**Published:** 2021-07-16

**Authors:** Sebastian Teichert, Martin G. J. Löder, Ines Pyko, Marlene Mordek, Christian Schulbert, Max Wisshak, Christian Laforsch

**Affiliations:** 1 GeoZentrum Nordbayern, Friedrich-Alexander-Universität Erlangen-Nürnberg (FAU), 91054 Erlangen, Germany; 2 Department of Animal Ecology I and BayCEER, University of Bayreuth, 95440 Bayreuth, Germany; 3 Marine Research Department, Senckenberg am Meer, 26382 Wilhelmshaven, Germany

**Keywords:** Ecology, Environmental sciences

## Abstract

There is an increasing number of studies reporting microplastic (MP) contamination in the Arctic environment. We analysed MP abundance in samples from a marine Arctic ecosystem that has not been investigated in this context and that features a high biodiversity: hollow rhodoliths gouged by the bivalve *Hiatella arctica*. This bivalve is a filter feeder that potentially accumulates MPs and may therefore reflect MP contamination of the rhodolith ecosystem at northern Svalbard. Our analyses revealed that 100% of the examined specimens were contaminated with MP, ranging between one and 184 MP particles per bivalve in samples from two water depths. Polymer composition and abundance differed strongly between both water depths: samples from 40 m water depth showed a generally higher concentration of MPs and were clearly dominated by polystyrene, samples from 27 m water depth were more balanced in composition, mainly consisting of polyethylene, polyethylene terephthalate, and polypropylene. Long-term consequences of MP contamination in the investigated bivalve species and for the rhodolith bed ecosystem are yet unclear. However, the uptake of MPs may potentially impact *H. arctica* and consequently its functioning as ecosystem engineers in Arctic rhodolith beds.

## Introduction

### Microplastics in marine ecosystems

Our modern daily life is largely founded on plastics—a diverse group of materials that can be designed to fit for a wide range of applications. The global production of plastics has steadily increased over the years and reached almost 360 million tons in the year 2018^1^. This in turn results in a growing number of plastic wastes worldwide. A significant part is carelessly discarded into the environment and finally often ends up in the oceans. It has been estimated that more than five trillion microplastic pieces and more than 250,000 tons of plastics are currently floating in the sea^2^ and that, e.g., in the year 2016, 19 to 23 million tons of new plastic waste entered aquatic environments^3^. Plastic particles are now reported in oceans worldwide^4^, from tropical^5^ to remote polar areas^6^, from beaches^7^ to deep sea sediments^8^.

Meanwhile, there is a great concern about the effects of plastics, especially regarding the so-called microplastics (MPs)^9^. Following the criteria developed by the US National Oceanic and Atmospheric Administration (NOAA), MPs refer to plastic items smaller than 5 mm^10^ and comprise a very heterogeneous group of particles that vary in polymer composition, additive content, size, shape, colour, origin, ageing state, and consequently their physicochemical properties^11^. The small size of MPs makes them potentially available for uptake by marine organisms especially at low trophic levels with the risk of food chain transfer^12^. Hence, a wide range of marine organisms has been documented to ingest MPs^13,14^. Especially bivalves and other filter feeders have a high potential susceptibility for the ingestion and bioaccumulation of MP particles^15^ and may therefore be used as sentinel organisms to monitor MP pollution^16^.

Regarding Arctic MPs, pollution data are still sparse in comparison with other regions of the world. So far, MPs have been found in Arctic waters from the sea surface through the water column to deep-sea sediments^17–20^, in considerable concentrations in Arctic sea ice^21,22^, in seawater beneath ice floes^23^, and in snow^24^. The ingestion of MPs has been reported for Arctic species, like planktivorous seabirds^25^, the juvenile polar cod *Boreogadus saida* (Lepechin, 1774)^26^, and several benthic species^27^ including bivalves^28^. However, highly biodiverse benthic communities of the Arctic subtidal zones—essential nursery grounds and important regions for commercial fishing^29^—have not been investigated for their MP contamination yet.

### The ‘reefs’ of the Arctic

Biodiversity in marine ecosystems depends on habitat heterogeneity, which is often increased by so-called ecosystem engineers^30^. One important benthic habitat generated by such ecosystem engineers are rhodolith beds, which can be found worldwide^31^. Generally, rhodoliths play an important role for the establishment and maintenance of marine biodiversity^32,33^ and their importance in providing ecosystem services like housing economically important species is well documented^34,35^. Rhodoliths are unattached calcite structures built by different species of coralline red algae. Their appearance ranges from small, massive cobbles to large, hollow spheres, thus with different effects on biodiversity^36^. In the Arctic, rhodolith beds are especially prominent around the Svalbard archipelago^37,38^, where they are built by coralline red algae mainly of the species *Lithothamnion glaciale* Kjellman, 1883. Many of those beds are dominated by hollow rhodoliths, which are generated by boring bivalves of the species *Hiatella arctica* (Linnaeus, 1767) that drill burrows into the rhodoliths which serves as protection against predators^39^. The suspension feeder^40^ *H. arctica* preferentially inhabits hard substrates and is a cosmopolitan species, found from pole to pole and from the intertidal zone down to 800 m water depth^41^. Within the Arctic, *H. arctica* is an important component of the benthic fauna in many coastal areas^42^. Usually, several of those filter-feeding bivalves inhabit one rhodolith and gouge it subsequently. It takes approximately 20 years until a bivalve-inhabited rhodolith becomes completely hollow. These hollow rhodoliths represent essential habitats for a wide range of benthic species, comparable to corals in tropical reefs^39^. Consequently, the provided habitat is a result of the interaction of two species. The rhodoliths themselves act as so-called autogenic ecosystem engineers, i.e. they change the environment by their own skeletal growth. *Hiatella arctica*, in this regard, acts as a so-called allogenic ecosystem engineer, i.e. it changes the environment by mechanical alteration of the rhodoliths. Only the interaction of both ecosystem engineers leads to the formation of the hollow rhodoliths around the Svalbard archipelago, and so increases the local biodiversity significantly^39^. The Svalbard rhodoliths are reported to shelter cnidarians (9 species), gastropods (10 species), bivalves (7 species), polychaetes (11 species), crustaceans (17 species), echinoderms (16 species), fishes (8 species), and many other groups^39^. Especially ophiuroids (brittlestars), which occur in great diversity and abundance^38^ constitute a major food source^43^ for the Arctic stock of cod, *Gadus morhua* Linnaeus, 1758. Kamenos et al.^44^ stated that due to the richness of food, rhodolith beds probably have a high holding capacity for juvenile gadoids, and are thus an important part of the inshore nursery system for commercial gadoid species like cod (*Gadus morhua*), saithe [*Pollachius virens* (Linnaeus, 1758)], and pollack [*Pollachius pollachius* (Linnaeus, 1758)]. This renders the rhodolith beds also potentially economically significant. Negative effects evoked by anthropogenic stressors on the bivalve-modified rhodolith microhabitat would thus also imply negative consequences for a wide range of associated species.

Among those stressors, like ocean acidification^45^ and global warming^46–48^, which may impact the rhodolith-bivalve system, are marine MPs. In fact, to date no data on the MP contamination of *H. arctica* in Arctic rhodolith beds are available. Our study aimed at a first estimation of the MP contamination of this species in Arctic rhodolith beds, which can be found around the Svalbard archipelago. Our samples were collected from two stations (27 m and 40 m water depth, respectively) at Mosselbukta, a bay situated at the northern coast of Spitsbergen, Svalbard archipelago. The hydrodynamics in this bay are complex^49^ and therefore might trigger different sedimentation regimes that also affect the pattern of MP distribution. Additionally, the size range of *H. arctica* specimens that are present in the rhodoliths strongly varies, so larger specimens might show higher MP concentrations due to possible accumulation effects. However, this would need to be analysed with respect to potential effects of the hydrodynamic regime. We investigated the MP accumulation in the bivalves by applying micro-computed tomography (µCT-3D) analysis for the identification of potential study specimens inside the rhodoliths, purification of the MP in the bivalve samples by a plastic-conserving enzymatic approach^50^, and identification of MP down to a size of ~ 10 µm in the samples via µFTIR-imaging and automated data analysis^51^.

It has already been shown that there is an increasing MP contamination in the Arctic environment. MPs can be transported via surface currents and bottom water transport over long distances^52^ and may segregate in the water column according to the density of their polymer types^53–55^. Our main hypotheses were (1) that there is a significant MP contamination in *H. arctica* in Arctic rhodolith beds of the Svalbard archipelago and, (2) that MP composition differs between both water depths due to a density dependent segregation in the water column, and (3) that MP concentration correlates to bivalve size due to potential accumulation effects.

## Results

Twelve bivalves (mean size in cm 2.36 ± 0.21 SE), assumed alive before preservation since the tissue was still present, were identified inside six rhodoliths via µCT-3D analysis. These bivalves were carefully extracted from the rhodoliths and the tissue of each bivalve was digested by enzymatic purification for subsequent µFTIR spectroscopy measurement and analysis.

We are aware that the present study reports on a limited number of bivalves. However, sampling in the high Arctic with a manned submersible is difficult and unfortunately restricts the number of samples which could be taken. Nevertheless, MP particles were detected in every single bivalve sample, ranging between one and 184 particles per bivalve after correction with the blank values. In total, 516 MP particles of two different morphotypes (89% fragments and 11% fibres) were found, comprising eight different polymer types: polyethylene (PE), polypropylene (PP), polystyrene (PS), polyethylene terephthalate (PET), polyamide (PA), polyvinyl chloride (PVC), polyacrylonitrile (PAN) and ethylene vinyl acetate (EVAC). Overall, 89% of particles belong to size class 4 (10–300 µm), 10% to size class 3 (300–1000 µm), and 1% to size class 2 (1000–5000 µm). There were no particles detected for size class 1 (> 5000 µm). Generally, most of the plastic types showed their maximum particle number in size class 4, except for PET, which was only found in size classes 2 and 3. Due to potential size specific filtration patterns in *H. arctica* and the methodology for identification (MPs > 10 µm), our findings do not allow to conclude on the whole size range of MPs which might be present in Mosselbukta. Despite the analysis of a limited number of samples, the detected size range of MPs is consistent throughout all bivalve samples. Results on MP polymer composition, blanks, and detailed data of the twelve bivalve samples—eight specimens from 27 m water depth and four specimens from 40 m water depth—are compiled in Supplementary Information Table 1.

**Figure 1:**
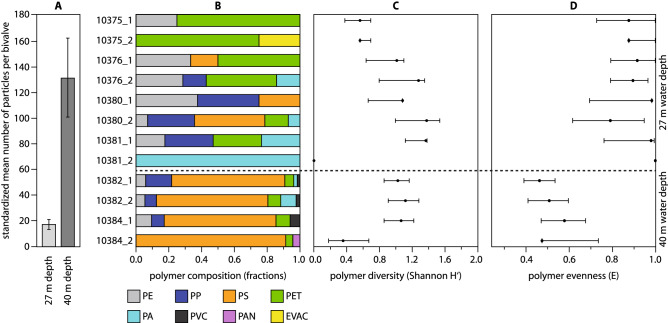
Abundances and diversities of microplastics. (**A**) Mean number of MP particles per bivalve (standardized to maximum shell length) at each sampling site. (**B**) Polymer composition in each bivalve; PE = polyethylene, PP = polypropylene, PS = polystyrene, PET = polyethylene terephthalate, PA = polyamide, PVC = polyvinyl chloride, PAN = polyacrylonitrile, EVAC = ethylene vinyl acetate, numbers from 10375_1 to 10384_2 correspond to *H. arctica* sample numbers used in Supplementary Information Table 1. (**C**) Polymer diversity (± SE) in each bivalve, indicating no significant differences between both sampling sites. (**D**) Polymer evenness (± SE) in each bivalve, indicating significantly lower evenness levels for the samples from the deeper sampling site.

The mean number of MP particles per bivalve at 27 m water depth was 8.1 ± 1.8 SE, being significantly lower than at 40 m water depth with 112.8 particles ± 33.4 SE (Kruskal–Wallis *H*(*chi*^2^) = 7.385, *H*_*C*_ (tie corrected) = 7.411, p(same) = 0.0065). This pattern was independent of bivalve size and persistent after standardization to maximum shell length (17.1 ± 4.0 SE at 27 m water depth, 131.5 ± 30.9 SE at 40 m water depth, Kruskal–Wallis *H*(*chi*^2^) = 6.490, *H*_*C*_ (tie corrected) = 6.536, p(same) = 0.011, see also Fig. 1A). With the exception of one bivalve, which contained only one PA particle, samples from 27 m water depth showed an inconsistent composition of polymers among samples. PET, PE, PP, PS, PA, and EVAC were found in different amounts (Fig. 1B). In contrast to that, samples from 40 m water depth were clearly dominated by PS. While the polymer EVAC is only found at 27 m water depth, PVC and PAN occurred only in samples from 40 m water depth (Fig. 1B). An evaluation of the polymer diversity for each sample based on the Shannon–Wiener-Index (H’) indicates no significant differences in terms of water depth (Kruskal–Wallis *H*(*chi*^2^) = 0.115, *H*_*C*_ (tie corrected) = 0.115, p(same) = 0.73, see also Fig. 1C). However, polymer type evenness based on the Buzas and Gibson index (e^H^/S) of samples from 27 m water depth is significantly higher than the evenness of samples from 40 m water depth (Kruskal–Wallis *H*(*chi*^2^) = 7.385, *H*_*C*_ (tie corrected) = 7.411, p(same) = 0.0065), which corresponds with the strong dominance of PS in the deeper samples (Fig. 1D).

**Figure 2:**
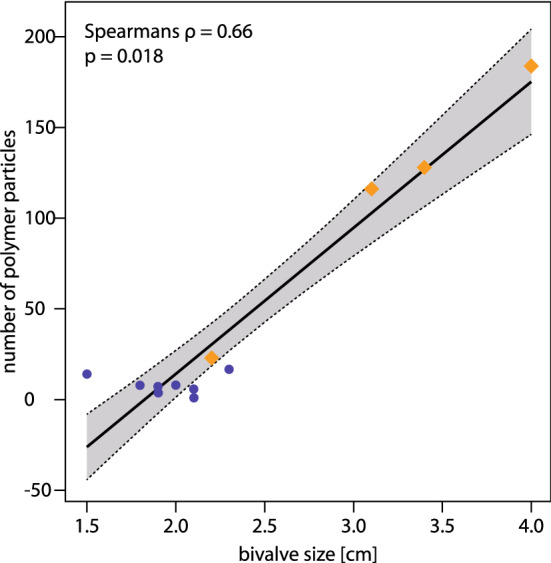
Significant positive correlation between bivalve size and number of MP particles of all size classes (Spearmans ρ = 0.66, *p* = 0.018). Violet circles indicate bivalve samples from 27 m water depth, orange diamonds indicate bivalve samples from 40 m water depth.

Independent of water depth, the amount of retained MP observed in our study significantly increases with increasing bivalve size (Spearmans ρ = 0.66, *p* = 0.018, Fig. 2). Results of the PERMANOVA analysis based on Euclidian distance indicate a significant effect of bivalve size on the number of retained particles (R^2^ = 0.92, *p* < 0.001) when coequally accounting for water depth as fixed factor and sampled rhodolith as random factor.

## Discussion

### MP contamination in *H. arctica* in comparison to other studies on marine bivalve species

A recent study on bivalves for human consumption from twelve different countries showed a worldwide contamination of bivalve tissue with MPs^56^. Our study is consistent with these findings, since all samples contained MP. However, a comparison between the rare data from studies on the MP contamination of free-living bivalves is generally difficult because of different analytical methods used and different size ranges analysed^57^. Despite this difficulty, the studies addressed for reasons of comparison are compiled in Table 1.

**Table 1:** Examples for MP contamination of different bivalve species.

Species	Locality	Contamination	Detection method	Analysed particle size range	References
*Crassostrea gigas*	North-West Atlantic	33%	Visual identification	> 500 µm	Rochman et al.^60^
*Mytilus galloprovincialis*	Mediterranean Sea	46.3%	FTIR	< 100–5000 µm (no lower limit stated)	Digka et al.^61^
*Mytilus galloprovincialis*	Mediterranean Sea	100%	µ-FTIR	20–100 µm	Gomiero et al.^63^
*Perna perna*	South-West Atlantic	75%	Visual identification	Not stated	Santana et al.^62^
*Mytilus* spp.	North Atlantic and Arctic Ocean	37.9% (analysed sites)	Pyrolysis GC/MS	> 10 µm (no upper limit stated)	Bråte et al.^28^
			µ-FTIR	> 50 µm (no upper limit stated)	
*Abra nitida*	North Atlantic	1.5%	µATR-FTIR Imaging	> 10 µm (no upper limit stated)	Bråte et al.^28^
*Limecola balthica*	North Atlantic and Baltic Sea	6.5%	µ-FTIR	> 50 µm (no upper limit stated)	Bråte et al.^28^
*Thyasira* spp.	North Atlantic	0%	µATR-FTIR Imaging	> 10 µm (no upper limit stated)	Bråte et al.^28^
*Hiatella arctica*	North Atlantic	0%	µ-FTIR	> 50 µm (no upper limit stated)	Bråte et al.^28^
*Hiatella arctica*	Arctic Ocean	100%	µ-FTIR Imaging	10–5000 µm	This study

In terms of sampling location (Svalbard, northern Norway) and target species (*H. arctica*), there is only one comparable study^28^. Our results are opposed to the findings of that large-scale survey on the MP contamination of five bivalve species—including *H. arctica*—across the Nordic marine environment. In that other study, *H. arctica* was the only included species not found to contain MPs. Bråte et al. 2020^28^ suggested that this was due to their very limited number of sampling sites (n = 3), covering a relatively small area at the coast of Norway. One reason for the contrasting results might be the different sampling location. However, a direct comparison with our data is difficult as Bråte et al. 2020^28^ did not analyse MPs smaller than 50 µm in their *H. arctica* samples. This is probably also the reason why they did not detect MPs in the only bivalve samples (*Mytilus* spp.) collected in Svalbard—our study area. However, their samples were collected inside Isfjorden, in an environment that is less influenced by major currents like the West Spitsbergen Current. Our samples, in contrast, derive from the comparatively exposed area of Mosselbukta, which is strongly impacted by the West Spitsbergen Current^58^. This is also in line with findings that exposed bays tend to show higher levels of MP contamination compared to sheltered bays, as for example in the interior of fjords^59^. A study on the Pacific oyster *Crassostrea gigas* (Thunberg, 1793) on the east coast of the United States found 33% of all specimens to be contaminated by MP^60^ and in the Northern Ionian Sea, 46.25% of all investigated Mediterranean mussels *Mytilus galloprovincialis* Lamarck, 1819 contained MP^61^. Only studies from highly urbanised areas such as the Santos estuary in Brazil—75% contamination rate of the brown mussel *Perna perna* (Linnaeus, 1758)^62^—or from coastal areas of the northern and central Adriatic Sea—100% contamination of the Mediterranean mussel *M. galloprovincialis* ^63^—showed as high contamination rates as found in our study. However, it has to be considered that we were only able to analyse a limited number of samples. In areas with a high anthropogenic impact such as the Adriatic Sea, a large semi-enclosed basin with a high accumulation potential for contaminants due to low water turnover rates, a high MP contamination rate in filter feeding organisms such as bivalves is expected. However, a standardisation of analytical methods for analysing MP in biota samples is urgently needed to ensure a better comparability of data.

With 8.1 (± 1.8 SE) particles per bivalve in 27 m water depth and 112.8 (± 33.4 SE) particles per bivalve in 40 m water depth, the absolute particle numbers are in line with other studies, ranging from 0.6 particles per specimen^60^ to 178 particles per specimen^64^. In relation to particle size, findings by Bråte et al. 2020^28^ showed that MPs detected in smaller species were generally smaller than the particles detected in larger species. Generally, Van Cauwenberghe et al. 2015^65^ showed that in bivalves, smaller particles are retained more frequently compared to larger particles. This is in concordance to our study since 89% of MP particles found in *H. arctica* were in the size range between 10 and 300 µm, thus representing the smallest investigated size class. The observed MP size pattern most probably relates to the species-specific size range of particles filtrated by *H. arctica*. Furthermore, due to the analytical method of micro-FTIR spectroscopy, MPs below 10 µm cannot be detected, and thus, our findings do only allow to estimate MP contamination of *H. arctica* in the size range of 10–5.000 µm.

Regarding the morphotypes of MPs in our bivalve samples, we found that fragments were most abundant (89%). This is in contrast to the results of a metanalysis by Li et al.^66^, which outlined that fibres are dominant in most of the current global field investigations on MPs in bivalves. However, it has to be mentioned that airborne fibre contamination is a frequent issue^67^ when handling samples in laboratories without contamination preventing facilities like laminar flow boxes. Whether the high abundance of fibres in bivalves found in the studies by Li et al. 2019^66^ is related to airborne sample contamination in the field and/or the laboratory or is due to a higher uptake of fibres is not known. Nevertheless, another recent study on the contamination of native *M. galloprovincialis* with MPs from the northern and central Adriatic Seas also showed a high abundance of fragments in both, coastal and offshore areas and therefore supports our findings^63^.

### Depth-specific MP composition in *H. arctica*

The sinking velocity of a MP particle is dependent on particle density, size and shape as well as fluid density and may be altered by weathering and biofouling^68^. MP particles may segregate in the water column according to the density of their polymer types^54,55^. The distribution of MP particles in our samples indicates a depth zonation of MP composition, with higher abundances of lower density polymers like PE in 27 m water depth and higher abundances of denser polymers like PS in 40 m water depth, which is corroborated by a lower evenness of polymer composition at the 40 m sampling spot. However, we found polymers with low and high density in both water depths, which is most probably caused by the general phenomenon of biofouling, which alters particle density and therefore vertical transport and distribution in the water column^69^. Additionally, the distribution patterns of MPs are of course also subject to local circulation features^20^, which are complex at Mosselbukta^58^ but due to the snapshot character of that study^58^, cannot be correlated to our findings with confidence. Li et al. 2019^66^ showed that the main morphotype and the polymer composition in bivalves tend to be consistent with the frequency of the different types of MPs in their surrounding environmental media. This renders bivalves as appropriate indicator organisms for MP contamination of a habitat. Assuming a correlation between findings of MP in bivalves and the surrounding water, we anticipate that the MP composition patterns in the bivalves mirror at least to a certain extent the MP-pollution in the different water depths within the Mosselbukta area.

### Potential origin of observed MPs

Previous studies indicate that the Arctic features sediments with a comparatively high degree of MP contamination^70^, possibly representing a global sink for plastic pollution^18^. Additionally, other studies have shown that in the context of climate change, the Arctic might also be a source of MPs via melting of Arctic sea ice containing a legacy of MP deposits from the past decades^21,22^ and that MPs can be found along the water column up to deep-sea sediments^20^. Our results indicate that the exposed subtidal Arctic ecosystem of Mosselbukta also exhibits a significant MP contamination.

However, tracking back the original sources of plastics debris and especially MPs is often difficult^71^ and this is of course also the case in the present study. In addition to local sources, long-range transport of MPs from temperate regions to the Arctic in both atmosphere and ocean currents are important pathways, but to date little understood. Other potential sources for MPs and plastic litter in the Arctic in general are fishery industries^20^ and the increasing number of tourist cruises^72^. However, transport with ocean currents from populated areas further south is likely. We hypothesize three major possible sources for the MPs in our study area: (1) sea ice, which acts as a temporary sink especially for MPs with strongly varying composition (always including PE and PA)^22^ and releases MPs during the increased melting caused by global warming^21^; (2) local plastic litter from the coast of Svalbard (80% fishing gear like nets and lines which consist mainly of nylon, i.e., PA)^72^; (3) floating plastics introduced by long range transports over ocean currents^6^. The observed different polymer composition patterns at the sampling stations with the observed depth differences in polymer distribution could however also be influenced by the small-scale hydrodynamic regime within the Mosselbukta area. The main sources for the water masses here are Atlantic water (transported by the West Spitsbergen Current), Arctic water, transformed Atlantic water, and polar surface water, whose particular impact in specific regions of Mosselbukta changes continuously and on the short-term^58^. Because of this variety of different water sources, it is not possible to determine their single contribution, i.e. which water mass is the main source of the MP particles detected in our samples.

### Potential ecological implications

A MP contamination of bivalve species from the Arctic has been shown before^28^, however, our data show that all bivalves retained a variety of MPs such as PE and PS. Also, larger bivalves contained higher numbers of MP particles, also when potentially varying distributions of MP particles in different water depths are considered. However, it cannot be discriminated if this is due to an accumulation effect within the bivalves or because of, e.g., a higher filtration rate of the larger bivalves. In general, the hypothetical pathways of MP intake and accumulation in bivalves are the following: When MPs in seawater encounter gill surfaces, they may be captured and trapped in mucus to be subsequently assimilated over the gill epithelium or to be transported into the digestive system, where they can translocate into the tissue^66,73^. Besides this process, MP adhesion to the soft tissue (mantle, gonad, adductor, visceral tissue, and foot) can further contribute to the MP presence of specimens^73,74^. Those processes might lead to a further increase of MPs in larger bivalves, which have higher filtration and uptake rates and thus consequently encounter more MPs than smaller ones. Due to the nature of the present study, the discrimination between the abovementioned aspects of MP contamination in bivalves could not be addressed, as we analysed the total MP contamination.

Ingestion of PS particles may influence reproduction and mortality^15^. For the ingestion of PE particles, histological changes and inflammatory responses are reported after translocation of the particles into the tissue of *M. edulis* ^73^. Indeed, our analytical methods do not enable the *in-situ* location of the MP particles within the bivalves, but increasing MP numbers towards the lower end of the detected MP size range suggests the presence of even smaller particles (< 10 µm) and a potential translocation of those MPs into the tissue of *H. arctica* ^65^ and negative effects on *H. arctica* cannot be excluded.

Since the rhodolith beds and the microhabitats supplied especially by the hollow rhodoliths are an important fundament for the local biodiversity around the Svalbard archipelago^39^, potential negative MP impacts on *H. arctica* may in turn affect the entire Arctic rhodolith bed ecosystem. If so, this would also affect local fish—including economically relevant species like the cod—which is known to use rhodolith beds as nursery grounds^44^.

Although we analysed a limited number of samples, our results show that the bivalve *H. arctica* from Arctic rhodolith beds is exposed to relatively high numbers of MPs already at present. We can anticipate that the contamination of the bivalves mirrors the environmental contamination in the Svalbard rhodolith beds, at least to a certain extent. This contamination state will however further deteriorate in the near future due to an ongoing input of plastics into the oceans and fragmentation of already present larger plastic items into MPs. If and how the increasing MP numbers in tandem with global change factors will affect the rhodolith bed ecosystem remains unclear, but potential future scenarios and the increasing anthropogenic threats—including rising temperatures, intensified fishery and tourist activities—are not promising.

## Materials and methods

### Collection of rhodoliths for bivalve sampling

**Figure 3:**
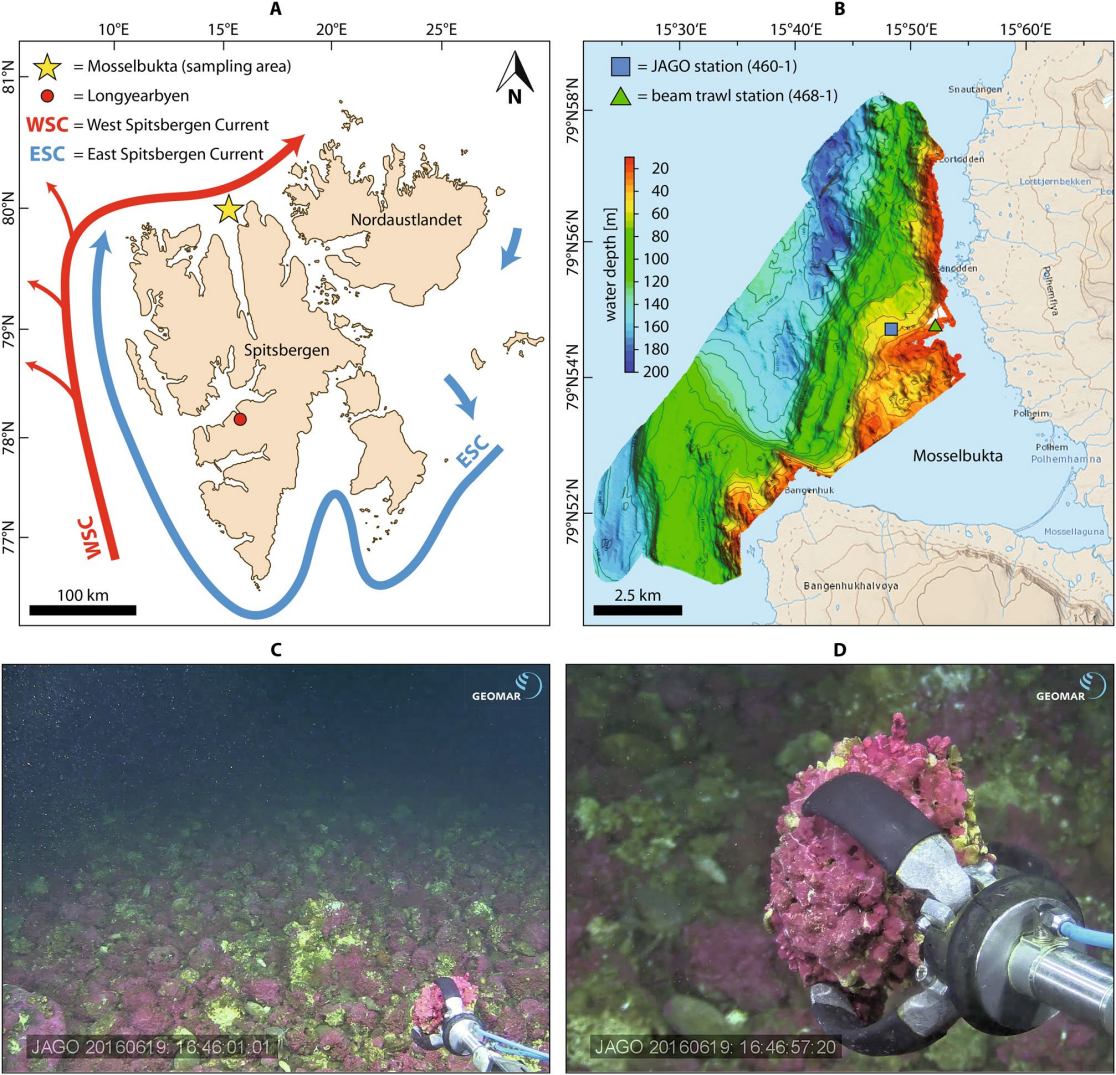
Rhodolith collection. (**A**) Location of the sampling area in Mosselbukta at the northern coast of Spitsbergen, Svalbard (topographic map used with courtesy of the Norwegian Polar Institute). (**B**) Multibeam bathymetric map of Mosselbukta indicating sampling station MSM55-468-1 (79° 54.80′ N, 15° 53.01′ E) in 27 m water depth, sampled by beam trawl and station MSM55-460-1 (79° 54.54′ N, 15° 48.41′ E) in 40 m water depth, sampled by JAGO. (**C**) Sampled rhodolith bed in Mosselbukta at station MSM55-460-1. (**D**) Manipulator arm of the JAGO submersible collecting a rhodolith at station MSM55-460-1.

Rhodoliths were obtained during the MSM55 expedition (ARCA) of RV *Maria S. Merian* from 11 to 29th of June 2016^49^. Samples were collected from two stations at Mosselbukta, a bay situated at the northern coast of Spitsbergen, Svalbard archipelago (Fig. 3A–C). One site (station MSM55-460-1, 79° 54.54′ N, 15° 48.41′ E) was sampled using the manned submersible JAGO in 40 m water depth (Fig. 3D), the other site (station MSM55-468-1, 79° 54.80′ N, 15° 53.01′ E) was sampled using a beam trawl in 27 m water depth. All rhodoliths were dried in cabinet desiccators at 30 °C for 48 h. At this point, rhodoliths in their distinctive hollow form can be considered as a sheltered system and protect the enclosed bivalves largely from outside MP contamination. Furthermore, the bivalves stop filtering and tightly close their shells as soon as the rhodoliths are recovered and fall dry on board of the research vessel. Normally, bivalve shells open after their death as a result of ligament relaxation, however, the desiccation process stiffened the ligament quickly, keeping the bivalves tightly closed as verified by visual inspection on board of *Maria S. Merian*. As a consequence, the bivalves had negligible contact with any laboratory equipment or air because they remained closed and sheltered inside the rhodolith skeleton. Because of this “double” protection of the bivalve tissue until further sample preparation, no blanks were used at this step. Dry rhodoliths were stored in sealed plastic bags (PE-LD) together with silica gel as drying agent (silica gel had no contact to the bivalves). Two rhodoliths (sample numbers SaM-10382 and SaM-10384) from station MSM55-460-1 and four rhodoliths (sample numbers SaM-10375, SaM-10376, SaM-10380, and SaM-10381) from station MSM55-468-1 fulfilling the before mentioned distinctive hollow form were chosen for the extraction of bivalve samples.

### Bivalve sampling

To localize the boring bivalves within the rhodoliths, the rhodolith specimens were scanned using micro-computed x-ray tomography (µCT). µCT scans were performed with a General Electric (GE)/Phoenix v|tome|x S 240. The µCT device was equipped with a GE x-ray source xs|240d with a tungsten target and a GE flat panel detector DXR250RT with 1000 × 1000 pixels. Scanning was done at a voltage of 140 kV and a current of 780 mA. A 1.0 mm copper filter was applied to reduce beam-hardening effects. For specimen 10376, which was smaller, field object distance (FOD) was 564 mm and voxel size was 13.8 µm. For all other rhodoliths, FOD was 580 mm and voxel size was 14.3 µm. The field detector distance (FDD) was 811 mm for all specimens.

**Figure 4:**
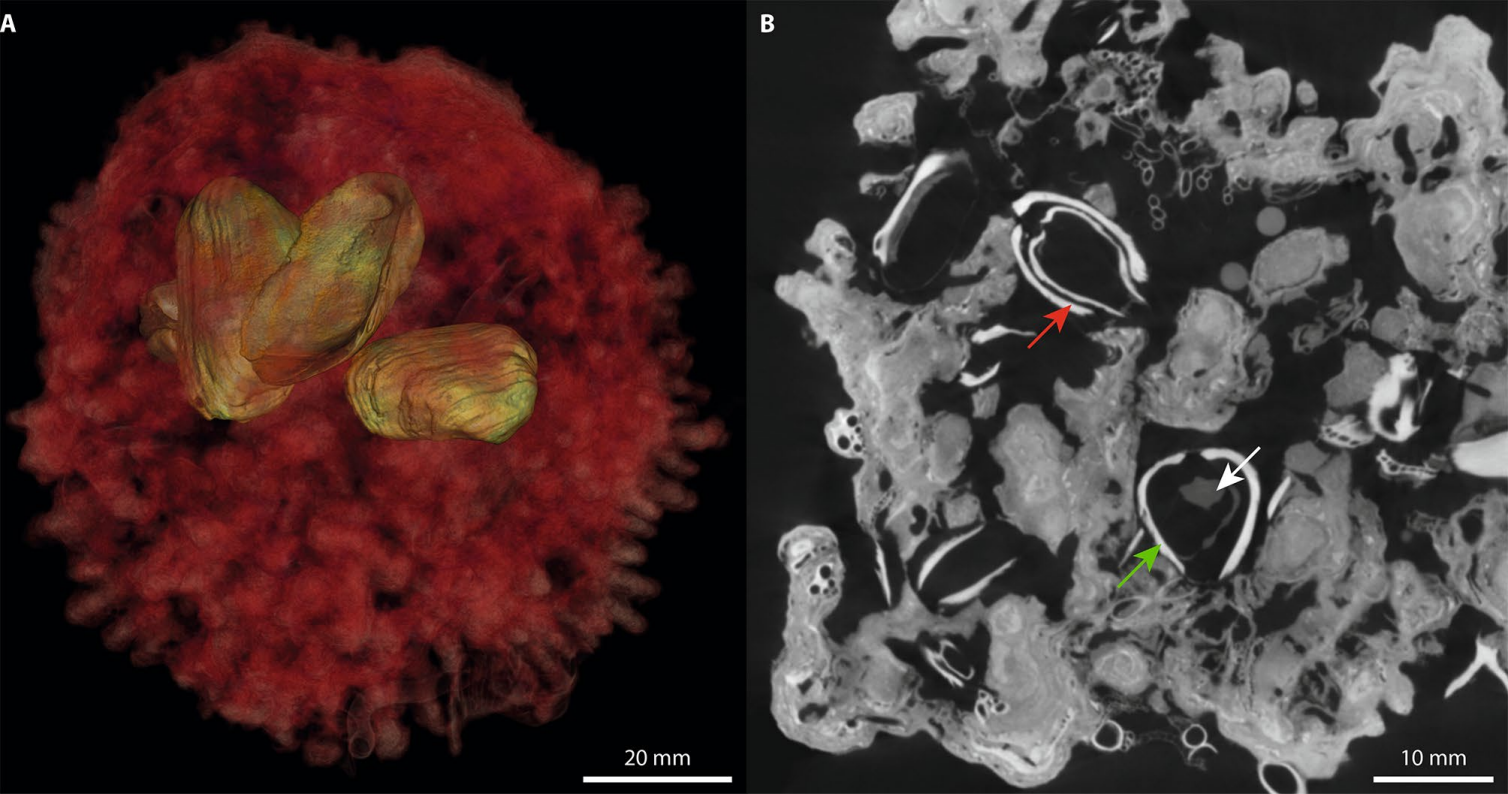
Bivalve sampling. (**A**) µCT-3D-reconstruction of a rhodolith from ca. 40 m water depth (SaM-10384, transparent red) including several specimens of *H. arctica* (green). (**B**) µCT-section of the same rhodolith (SaM-10384) with specimens of *H. arctica* that were dead (red arrow) and living (green arrow) at the time of sampling; note soft tissue visible in dark grey within bivalves alive at sampling time (white arrow).

Raw data were reconstructed and merged with GE datos|x software version 2.4, using a Feldkamp algorithm based on filtered back projection. For noise reduction of the reconstructed data set, a median filter (VolumeGraphics) within a local neighbourhood of 3 voxels (diameter of neighbourhood area) was used. All post-processing was performed with the VolumeGraphics Studio MAX software version 3.0. Rhodoliths and bivalves were segmented mainly manually. The segmented datasets were inspected visually and detected bivalves containing sufficient amounts of soft tissue—indicating that the mussels were alive during sampling—were selected for extraction (Fig. 4A, B). Rhodoliths were opened using a metal hammer and a metal chisel and two bivalves per rhodolith (numbered 10375-1 and 10375-2, etc.) were extracted with metal tweezers. Longitudinal shell length of the bivalves was measured using the software CellSense (Olympus) and the bivalves were wrapped in aluminium foil for temporary storage.

### General laboratory precautions for microplastic analyses

Plastic-fibre free lab coats were worn in the laboratory and work surfaces were precleaned and dust-free prior to working. Used laboratory equipment was plastic-free wherever possible and cleaned before usage by filtered ethanol (35%, 0.2 µm Whatman ME 24 membrane filters) and demineralised water (0.2 µm and 0.45 µm Sartoban 300 Sterile Capsules). All chemicals used were filtered before usage applying 0.45 µm Whatman RC 55 membrane filters. Reagents were stored in glass bottles and always covered by aluminium foil.

### Purification of bivalve tissue

The protocol for purification used here was an adjusted version of the methodology suggested by Löder et al. 2017^50^. The dried soft tissue of each specimen was rehydrated using sodium dodecyl sulphate solution (SDS, 10%) at 50 °C for three weeks in small glass beakers with glass lid (J. Weck GmbH u. Co. KG). The tissue was then removed from the shells, sheared and treated with SDS solution (10%) at 50 °C for further six days. Afterwards, the tissue was digested using an enzymatic-oxidative seven-step process in Weck glass beakers in incubators, if not stated otherwise:

1. 10 ml protease, 25 ml Tris-buffer at pH 9 and 40 °C for 3 days
2. Rerun of step (1)
3. 1 ml lipase, 25 ml Tris-buffer at pH 9 and 40 °C for 24 h
4. 5 ml cellulase, 25 ml acetate buffer at pH 5 and 40 °C for 24 h
5. 30 ml H_2_O_2_ (30%) at 37 °C for 24 h (two times consecutively)
6. 1 ml chitinase, 25 ml acetate buffer at pH 5 and 37 °C for 4 days
7. Catalytic wet-peroxide oxidation (two times consecutively under a fume hood): 20 ml H_2_O_2_ (30%), 20 ml 0,05 M Fe (II) solution, stirred in glass beaker with thermometer and heated to 30 °C, cooled in an ice bath if the exothermic reaction led to a temperature rise above 60 °C. Colour change from amber to yellow indicated end of reaction time.

Between all steps, the sample material was filtered using 10 µm stainless steel filters in a stainless-steel filtration unit (Sartorius AG). The H_2_O_2_ steps were used to remove proteins protecting organismic chitin structures for a better efficiency of the following chitinase step and were repeated to increase the efficiency with fresh H_2_O_2_ solution. The purification process was very effective and only negligible rests of organic material remained. For a detailed description of the enzymatic-oxidative purification process, please compare Löder et al. 2017^50^.

### Size fractionation, density separation and spectroscopic analysis of the purified samples

After the last purification step the purified material was rinsed off from the stainless-steel filter and wet size-fractionated using a 500 µm stainless steel sieve resulting in two fractions (< 500 µm and > 500 µm, respectively).

Potential MP particles > 500 µm were sorted out by hand under a stereomicroscope, photo documented, size measured and then analysed with attenuated total reflectance (ATR) FTIR spectroscopy on a Bruker ALPHA ATR unit equipped with a diamond ATR crystal. The FTIR measurement was conducted in a wavenumber range of 4000–400 cm^−1^ with a resolution of 8 cm^−1^ and eight accumulated scans. The background was measured against air with the same parameters. The polymer origin of potential MP particles was identified by comparison of the sample spectra with a self-generated polymer library^75^. For QA/QC all measured sample spectra were double-checked by experienced personnel.

The fraction < 500 µm was dehydrated using ethanol (98%) prior to density separation to remove mineral residues using zinc chloride solution (1.7 g cm^−3^) in separatory funnels (compare Löder et al. 2017^50^). After density separation, the whole sample of the particle fractions < 500 µm was filtered on Anodisc filters (25 mm diameter, 0.2 µm pore size, Whatman) with the help of a custom-made glass filter holder resulting in a round sample area of approximately 10 mm diameter on the filters. Filters were placed on calcium fluoride IR windows for measurement by FTIR chemical imaging.

FTIR chemical imaging for quantitative and qualitative analysis of the filter containing the particles < 500 µm was conducted with focal plane array (FPA) detector-based micro-Fourier-transform infrared (micro-FTIR) spectroscopy: FTIR spectra of the whole sample area were recorded using a Bruker HYPERION 3000 FTIR microscope equipped with a 64 × 64 detector pixel FPA and coupled to a TENSOR 27 spectrometer. After collection of a visual overview image with a 4 × objective lens FTIR spectra were measured with a 15 × IR cassegrain objective lens in a wavenumber range of 3600–1250 cm^−1^ with a resolution of 8 cm^−1^, six accumulated scans and 4 × 4 binning, resulting in a pixel size of ca. 10 µm. The background was measured on the pure filter surface with the same parameters and 32 accumulated scans. Measurements were operated with the Bruker OPUS software version 7.5. Data of the chemical images were imported into the software Image Lab version 4.1 (EPINA GmbH) in .envi format and the automated analysis of the whole sample was performed with the ‘BayreuthParticleFinder’ module based on random decision forest classifiers^51^ for the eleven most important plastic types. The software includes an automated particle size measurement. Again, for QA/QC all particles automatically classified as plastic particles were double-checked according to their IR spectra by experienced personnel.

The plastic particle identified by ATR-FTIR spectroscopy (particle size > 500 µm) and FTIR chemical imaging (particle size < 500 µm) could be assigned to the eight polymer classes polyethylene (PE), polypropylene (PP), polystyrene (PS), polyethylene terephthalate (PET), polyamide (PA), polyvinyl chloride (PVC), polyacrylonitrile (PAN), ethylene vinyl acetate (EVAC) (no other polymers were found) and were sorted into size classes according to their longest dimension. Size class 4 corresponds to 10–300 µm, size class 3 corresponds to 300–1000 µm, size class 2 corresponds to 1000–5000 µm, and size class 1 corresponds to macroplastics (> 5000 µm).

To account for potential contamination with MP particles during laboratory work, four blank samples using demineralised, filtered water were co-processed with the samples. During data procession, mean values of particle numbers obtained from the four blank samples for each polymer and size class were rounded up to the next integer in the sense of a conservative approach and were subtracted from the respective polymer and size class in every sample.

In the blanks, a higher contamination with PET particles only in the smallest size class 4 was found and subtracted from the samples (Supplementary Information Table 2). However, in the bivalve samples PET particles were additionally found in size class 3 and 2 and thus were counted as retained MP particles as bivalves were closed at the moment of sampling and were located within a rhodolith, so contamination issues should be if at all of negligible importance as described above.

### Statistical data analysis

All statistical analyses were performed in PAST version 3.25^76^ and in R version 3.5.2^77^. Since rhodoliths only act as substrate and bivalves filter the water surrounding the rhodoliths, the bivalves represent the experimental units in our study. To compare the mean numbers of MP per bivalve in each water depth, particle numbers were standardized to maximum bivalve shell length. For each bivalve sample, polymer fractions were calculated and polymer composition was characterized utilizing the Shannon–Wiener-Index (H’) as well as the evenness based on the Buzas and Gibson index (e^H^/S). To estimate if there are significant differences between both water depths regarding particle numbers and polymer composition, we used the non-parametric Kruskal–Wallis test because the prerequisites for parametric tests, i.e., homogeneity of variance and normality, were not given. Additionally, the Kruskal–Wallis test is applicable for unbalanced sample designs^78^ (eight specimens from 27 water depth vs. four specimens from 40 m water depth). To account for the relationship between bivalve size and the number of retained polymer particles, we used Spearman rank correlation. It has to be considered, however, that samples from 27 m water depth are dominated by smaller bivalves and samples from 40 m water depth are dominated by larger bivalves (see Supplementary Information Table 1). Additionally, two bivalves were taken from one rhodolith, each, so rhodoliths have to be considered a random factor. To account for this, we performed a PERMANOVA based on Euclidian distance with an intercept varying among sites (depth) and among blocks (rhodoliths) within sites (nested random effects model) using the “adonis” function in the R-package “vegan” version 2.5-7^79^, coding as

$${\text{adonis}}\left( {{\text{particles}}\sim {\text{size}},\;{\text{strata}} = 1|{\text{depth}}/{\text{rhodolith}},\;{\text{permutations}} = 999,\;{\text{method}} = \,``{\text{euclidian''}}} \right)$$

where “particles” represents the number of MP particles detected in each bivalve (numeric), “size” the body size of each bivalves (numeric) “depth” the water depth from which the sample derives (numeric), and “rhodolith” the rhodoliths from which two bivalves have been sampled each (factor).

## Supplementary Information

Supplementary Information.
