# Supplementary material for: Microplastic contamination of the drilling bivalve Hiatella arctica in Arctic rhodolith beds
Source: Sci Rep. 2021 Jul 16;11:14574. doi: 10.1038/s41598-021-93668-w (PMC8285507; doi:10.1038/s41598-021-93668-w)
Supplement: Supplementary file 1 — Supplementary Information. [file 41598_2021_93668_MOESM1_ESM.pdf]

Supplementary Information Table 1+2 | Sampling data and polymer composition in bivalve and blank samples

Information on the sampling sites, methods, and size of each bivalves as well as on the analysed composition and size classes of ingested MP particles after subtraction of the blank values (Table 1); mean values of MP particles found in four blank samples rounded up to the next integer (Table 2); PE = polyethylene, PP = polypropylene, PS = polystyrene, PET = polyethylene terephthalate, PA = polyamide, PVC = polyvinyl chloride, PAN = polyacrylonitrile, EVAC = ethylene vinyl acetate; size class 2 = 1,000 – 5,000 µm, size class 3 = 300 – 1,000 µm, size class 4 = 10 – 300 µm.

Supplementery Table 1

bivavles	polymer types										bivalve size [cm]	sampling data				
sample ID	*particle sizeclass*	*PE*	*PP*	*PS*	*PET*	*PA*	*PVC*	*PAN*	*EVAC*	*total number of particles*		*depth [m]*	*station*	*coordinates*	*sampling date*	*sampling gear*
**SaM-10375_1**	2	0	0	0	0	0	0	0	0	0						
	3	0	0	0	6	0	0	0	0	6						
	4	2	0	0	0	0	0	0	0	2						
	total	**2**	**0**	**0**	**6**	**0**	**0**	**0**	**0**	**8**	2.0	27	MSM55-468-1	79°54.80'N, 15°53.01'E	20th June 2016	beam trawl
**SaM-10375_42**	2	0	0	0	0	0	0	0	0	0						
	3	0	0	0	3	0	0	0	1	4						
	4	0	0	0	0	0	0	0	0	0						
	total	**0**	**0**	**0**	**3**	**0**	**0**	**0**	**1**	**4**	1.9	27	MSM55-468-1	79°54.80'N, 15°53.01'E	20th June 2016	beam trawl
**SaM-10376_1**	2	0	0	0	0	0	0	0	0	0						
	3	0	0	0	3	0	0	0	0	3						
	4	2	0	1	0	0	0	0	0	3						
	total	**2**	**0**	**1**	**3**	**0**	**0**	**0**	**0**	**6**	2.1	27	MSM55-468-1	79°54.80'N, 15°53.01'E	20th June 2016	beam trawl
**SaM-10376_2**	2	0	0	0	0	0	0	0	0	0						
	3	0	0	0	3	0	0	0	0	3						
	4	2	1	0	0	1	0	0	0	4						
	total	**2**	**1**	**0**	**3**	**1**	**0**	**0**	**0**	**7**	1.9	27	MSM55-468-1	79°54.80'N, 15°53.01'E	20th June 2016	beam trawl
**SaM-10380_1**	2	0	0	0	0	0	0	0	0	0						
	3	0	0	0	0	0	0	0	0	0						
	4	3	3	2	0	0	0	0	0	8						
	total	**3**	**3**	**2**	**0**	**0**	**0**	**0**	**0**	**8**	1.8	27	MSM55-468-1	79°54.80'N, 15°53.01'E	20th June 2016	beam trawl
**SaM-10380_2**	2	0	0	0	0	0	0	0	0	0						
	3	0	0	0	2	0	0	0	0	2						
	4	1	4	6	0	1	0	0	0	12						
	total	**1**	**4**	**6**	**2**	**1**	**0**	**0**	**0**	**14**	1.5	27	MSM55-468-1	79°54.80'N, 15°53.01'E	20th June 2016	beam trawl
**SaM-10381_1**	2	0	0	0	3	0	0	0	0	3						
	3	0	0	0	2	0	0	0	0	2						
	4	3	5	0	0	4	0	0	0	12						
	total	**3**	**5**	**0**	**5**	**4**	**0**	**0**	**0**	**17**	2.3	27	MSM55-468-1	79°54.80'N, 15°53.01'E	20th June 2016	beam trawl
**SaM-10381_2**	2	0	0	0	0	0	0	0	0	0						
	3	0	0	0	0	0	0	0	0	0						
	4	0	0	0	0	1	0	0	0	1						
	total	**0**	**0**	**0**	**0**	**1**	**0**	**0**	**0**	**1**	2.1	27	MSM55-468-1	79°54.80'N, 15°53.01'E	20th June 2016	beam trawl
**SaM-10382_1**	2	0	0	0	0	0	0	0	0	0						
	3	0	0	4	10	0	0	0	0	14						
	4	11	29	123	0	4	3	0	0	170						
	total	**11**	**29**	**127**	**10**	**4**	**3**	**0**	**0**	**184**	4.0	40	MSM55-460-1	79°54.54'N, 15°48.41'E	19th June 2016	JAGO
**SaM-10382_2**	2	0	0	0	0	0	0	0	0	0						
	3	0	0	0	10	0	0	0	0	10						
	4	7	9	87	0	12	3	0	0	118						
	total	**7**	**9**	**87**	**10**	**12**	**3**	**0**	**0**	**128**	3.4	40	MSM55-460-1	79°54.54'N, 15°48.41'E	19th June 2016	JAGO
**SaM-10384_1**	2	0	0	0	0	0	0	0	0	0						
	3	0	0	0	10	0	0	0	0	10						
	4	11	9	79	0	0	7	0	0	106						
	total	**11**	**9**	**79**	**10**	**0**	**7**	**0**	**0**	**116**	3.1	40	MSM55-460-1	79°54.54'N, 15°48.41'E	19th June 2016	JAGO
**SaM-10384_2**	2	0	0	0	1	0	0	0	0	1						
	3	0	0	0	0	0	0	0	0	0						
	4	0	0	21	0	0	0	1	0	22						
	total	**0**	**0**	**21**	**1**	**0**	**0**	**1**	**0**	**23**	2.2	40	MSM55-460-1	79°54.54'N, 15°48.41'E	19th June 2016	JAGO

Supplementery Table 2

**Mean values of MP particles found in four blank samples (rounded up to the next integer)**	polymer types								
	particle sizeclass	*PE*	*PP*	*PS*	*PET*	*PA*	*PVC*	*PAN*	*EVAC*
	1	*0*	*0*	*0*	*0*	*0*	*0*	*0*	*0*
	2	0	0	0	1	0	0	0	0
	3	0	1	0	2	0	0	1	0
	4	1	7	1	70	0	1	0	0
